# Pressure-dependent stress relaxation in acute respiratory distress syndrome and healthy lungs: an investigation based on a viscoelastic model

**DOI:** 10.1186/cc8203

**Published:** 2009-12-09

**Authors:** Steven Ganzert, Knut Möller, Daniel Steinmann, Stefan Schumann, Josef Guttmann

**Affiliations:** 1 Department of Anesthesiology and Critical Care Medicine, University Medical Center, Freiburg, Hugstetter Str. 55, D-79106 Freiburg, Germany; 2 Department of Biomedical Engineering, Furtwangen University, Villingen-Schwenningen Campus, Jakob Kienzle Str. 17, D-78054 Villingen-Schwenningen, Germany

## Abstract

**Introduction:**

Limiting the energy transfer between ventilator and lung is crucial for ventilatory strategy in acute respiratory distress syndrome (ARDS). Part of the energy is transmitted to the viscoelastic tissue components where it is stored or dissipates. In mechanically ventilated patients, viscoelasticity can be investigated by analyzing pulmonary stress relaxation. While stress relaxation processes of the lung have been intensively investigated, non-linear interrelations have not been systematically analyzed, and such analyses have been limited to small volume or pressure ranges. In this study, stress relaxation of mechanically ventilated lungs was investigated, focusing on non-linear dependence on pressure. The range of inspiratory capacity was analyzed up to a plateau pressure of 45 cmH_2_O.

**Methods:**

Twenty ARDS patients and eleven patients with normal lungs under mechanical ventilation were included. Rapid flow interruptions were repetitively applied using an automated super-syringe maneuver. Viscoelastic resistance, compliance and time constant were determined by multiple regression analysis using a lumped parameter model. This same viscoelastic model was used to investigate the frequency dependence of the respiratory system's impedance.

**Results:**

The viscoelastic time constant was independent of pressure, and it did not differ between normal and ARDS lungs. In contrast, viscoelastic resistance increased non-linearly with pressure (normal: 8.4 (7.4-11.9) [median (lower - upper quartile)] to 35.2 (25.6-39.5) cmH_2_O·sec/L; ARDS: 11.9 (9.2-22.1) to 73.5 (56.8-98.7)cmH_2_O·sec/L), and viscoelastic compliance decreased non-linearly with pressure (normal: 130.1(116.9-151.3) to 37.4(34.7-46.3) mL/cmH_2_O; ARDS: 125.8(80.0-211.0) to 17.1(13.8-24.7)mL/cmH_2_O). The pulmonary impedance increased with pressure and decreased with respiratory frequency.

**Conclusions:**

Viscoelastic compliance and resistance are highly non-linear with respect to pressure and differ considerably between ARDS and normal lungs. None of these characteristics can be observed for the viscoelastic time constant. From our analysis of viscoelastic properties we cautiously conclude that the energy transfer from the respirator to the lung can be reduced by application of low inspiratory plateau pressures and high respiratory frequencies. This we consider to be potentially lung protective.

## Introduction

In the 1990s, low tidal volume and pressure-limited ventilation were supposed to lower mortality in patients mechanically ventilated for acute respiratory distress syndrome (ARDS) [1]. In a way, this was the beginning of lung-protective ventilation strategies [2]. Since then, a variety of such strategies targeting the reduction of ventilator-associated lung injury has been proposed [3-5]. A prerequisite for these developments is the knowledge about mechanical interactions within the respiratory system under the condition of mechanical ventilation.

During mechanical ventilation, energy is transferred from the ventilator to the patient's respiratory system. As in volutrauma and barotrauma, the amount of transferred energy is directly related to ventilator associated lung injury. However, volutrauma and barotrauma are both restricted to the particular physical quantities volume and pressure. Other parameters also directly influencing the transferred energy as the respiratory rate [6] are disregarded in these concepts. One could subsume all those different factors under an energy-related concept of lung injury. Hence, minimizing this 'energo-trauma' would be equivalent to the minimization of energy transfer by simultaneously adapting pressure, volume and frequency. This could be helpful in the development of lung-protective ventilation strategies.

One part of the transferred energy is required to overcome respiratory system resistance and compliance, another part is stored or dissipates in the viscoelastic components of the respiratory system while following the respiratory cycle. Exposing the lung tissue to an abrupt change in volume causes a stress relaxation response, which is a power function of time and depends on the viscoelastic properties of the respiratory system. Such stress relaxation curves can be obtained using methods based on the interrupter technique [7-9]. By the sudden interruption of (inspiratory) airflow, the respiratory pressure instantaneously drops by the amount of the resistive pressure fraction (airflow rate immediately preceding flow interruption multiplied by the Newtonian resistance of the respiratory system). This initial drop in pressure is followed by a slow decrease in pressure [10], which is caused by stress relaxation processes. Different mathematical models have been developed to interpret the associated physiological mechanisms [11,12].

During the past few decades, the effects of stress relaxation caused by the viscoelastic properties of lung tissue have been intensively investigated by model-based analysis techniques [13-24]. In these studies, viscoelastic parameters were usually assumed to be constant. However, Eissa and colleagues [18] found that this assumption holds true only for the baseline tidal volume range on zero end-expiratory pressure (ZEEP) and up to applied volumes of 0.7 L. It was speculated that this might reflect non-linear viscoelastic behavior for higher pulmonary volumes. In addition, Sharp and colleagues [13] reported that when inflating normal lungs with successive steps of equal volume (0.5 L), up to a final volume of 3.0 L, the amplitude of the slow pressure drop owing to stress adaptation increases non-linearly with inflation volume. However, the approaches applied in these studies were not specifically designed to quantify such non-linear effects or their progression over wide ranges of pressure and volume. Moreover, the dynamic loading process during volume inflation has not been taken into account because parameter estimation has been exclusively based on the stress relaxation curves under static zero-flow conditions.

The purpose of the present study was to investigate non-linear pressure-dependent viscoelastic properties of the respiratory system with focus on differences in energy distribution between healthy and ARDS lungs. The total range of inspiratory capacity was analyzed up to a plateau pressure of 45 cmH_2_O. The analysis included both the processes of dynamic loading and static stress relaxation of the tissue. For data acquisition, standardized super-syringe maneuvers were automatically performed. Data analysis was based on a viscoelastic lumped parameter model. Frequency related characteristics were investigated by impedance analysis.

## Materials and methods

### Patients and mechanical ventilation

The datasets for this retrospective study were obtained from two patient studies: (i) a multicenter study including 28 mechanically ventilated ARDS patients [25,26] (ARDS group); and (ii) a study including 13 mechanically ventilated patients under conditions of preoperative anesthesia [27] (control group). Data from super-syringe maneuvers were available from 20 of 28 patients (ARDS group) and from 11 of 13 patients (control group). Data for this retrospective study were obtained from two clinical trials. As the registration of clinical trials has been recommended for the beginning of 2008 and has been required since January 2009 these studies were not registered as having been performed before. Both patient studies (ARDS group, control group) were approved by the local ethics committees. Written informed consent was obtained from patients, next of kin or a legal representative. Automated respiratory maneuvers were applied using identical equipment (Evita4Lab-system, Dräger Medical, Lübeck, Germany). Gas flow was measured using a pneumotachograph (Fleisch No. 2, F+G GmbH, Hechingen, Germany). Volume was determined by integration ofthe flow signal. Airway pressure was measured using a differential pressure transducer (PC100SDSF, Hoffrichter, Schwerin, Germany). Flow and pressure data were measured proximally to the endotracheal tube at a sampling rate of 125 Hz. Patients were ventilated in the volume-controlled mode and at a constant inspiratory flow rate.

### Subjects and medication of ARDS group

Data were collected in the context of a multicenter study, which was carried out in intensive care units across eight German university hospitals. *Patients*: Patients suffering from pulmonary (n = 5) or extrapulmonary (n = 15) ARDS were included in the study. Patients had to be mechanically ventilated for 24 hours or longer before entering the study. *Exclusion criteria *were: patients considered ready to be weaned by the attending physician; in the terminal stage of disease; the presence of an obstructive lung disease, a bronchopleural fistula or known air leakage; hemodynamic instability or intolerance to a five minute ZEEP phase; age below 16 years; or pregnancy. *Medication*: Neuromuscular blocking drugs were applied as required. Sedatives were administered to achieve a Ramsay sedation score of 4 to 5. *Ventilation*: Patients were ventilated in a volume-controlled mode with a constant inspiratory flow rate in the supine position. The tidal volume was targeted at 8.0 ± 2.0 mL/kg. Inspiratory time and flow rate were set to obtain an end-inspiratory hold of 0.2 seconds or longer. Before the measurements, respiratory rate was adjusted to keep the partial pressure of arterial carbon dioxide below 55.0 mmHg. Between respiratory maneuvers, the fraction of inspired oxygen (FiO_2_) was chosen to maintain arterial oxygen saturation above 90%. *Maneuvers*: During the protocol, ventilator settings remained unchanged. During respiratory maneuvers, the FiO_2 _was set to 1.0. Five different maneuvers (low-flow inflation [28], incremental positive end-expiratory pressure trial (PEEP wave [29]), enlarged tidal volume breath for dynamic pressure-volume analysis (SLICE method [30]), static compliance by automated single steps [31] and super-syringe [32]) were performed in random sequence. To obtain standard volume history, patients were ventilated with ZEEP for five minutes before each maneuver. See Table 1 for details.

**Table 1:** Characteristics of ARDS group

Number	Weight (kg)	Primary diagnosis	ARDS (p/ep)
1	**125**	**Pancreatitis**	**ep**
2	**100**	**Severe thorax trauma**	**p**
3	**65**	**Pancreatitis**	**ep**
4	**85**	**Peritonitis**	**ep**
5	**61**	**Peritonitis**	**ep**
6	**100**	**Pneumonia**	**p**
7	**90**	**Traumatic open brain injury**	**ep**
8	**104**	**Postresuscitation after heart failure**	**ep**
9	**60**	**Peritonitis**	**ep**
10	**70**	**Subarachnoid hemorrhage**	**ep**
11	**85**	**Peritonitis**	**ep**
12	**85**	**Traumatic brain injury**	**ep**
13	**80**	**Carcinoma of the floor of the mouth**	**ep**
14	**95**	**Pneumonia**	**p**
15	**75**	**Traumatic brain injury**	**ep**
16	**63**	**Pneumonia**	**p**
17	**66**	**Abdominal aortic aneurysm**	**ep**
18	**90**	**Pancreatitis**	**ep**
19	**90**	**Pneumonia after blunt abdominal trauma**	**p**
20	**70**	**Liver cirrhosis**	**ep**

Mean	**87.7**		
SD	**28.5**		

ARDS = acute respiratory distress syndrome; ep = extra-pulmonary; p = pulmonary; SD = standard deviation.

### Subjects and medication of control group

Data was measured under conditions of preoperative anesthesia for orthopedic surgery at the University Hospital of Freiburg. *Patients*: Patients in American Society of Anesthesiologists' (ASA) physical status I and II undergoing general anesthesia and tracheal intubation were included in the study. *Exclusion criteria *were: patients with indications of lung disease; age below 18 years; as electrical impedance tomography was also performed in these patients (data not used in this study), the presence of any condition precluding the implementation of electrical impedance tomography such as a pacemaker, an implanted automatic cardioverter defibrillator, implantable pumps, pregnancy, lactation period, or iontophoresis. *Medication*: Anesthesia was induced with fentanyl and propofol. Propofol was applied continuously to maintain anesthesia. Vecuronium bromide was applied for neuromuscular blocking. *Ventilation*: Patients were ventilated in the volume-controlled mode (10 mL/kg, respiratory rate 12 breaths/minute, inspiratory:expiratory ratio: 1:1.5, FiO_2_: 1, PEEP 0 cmH_2_O) while in the supine position. To prevent potential atelectasis, a recruitment maneuver was performed by increasing PEEP up to a plateau pressure of 45 cmH_2_O. Ventilation at the corresponding PEEP was maintained for six breaths and then reduced to ZEEP. *Maneuvers*: An incremental PEEP trial [29] followed by a super-syringe maneuver [32] was performed. To standardize volume history, both maneuvers were preceded by ventilation with ZEEP for five minutes. See Table 2 for details.

**Table 2:** Characteristics of control group

Number	Weight (kg)	Primary diagnosis
1	**64**	**Lesion of the right anterior meniscus**
2	**83**	**Rupture of right anterior cruciate ligament**
3	**68**	**State after fracture of left upper arm**
4	**85**	**Bimalleolar ancle joint fracture**
5	**85**	**Compartment syndrome left lower leg**
6	**87**	**Cartilage damage medial condyle of femur**
7	**89**	**Fracture of left lateral tibial plateau**
8	**72**	**State after plating of fractured olecranon**
9	**77**	**Bilateral fracture of lower leg, fractured left ancle joint**
10	**70**	**Four-part fracture of head of humerus**
11	**63**	**Lesion of ventral capsule-labrum-complex of right shoulder**

Mean	**76.7**	
SD	**9.6**	

SD = standard deviation.

### Datasets

**Figure 1:**
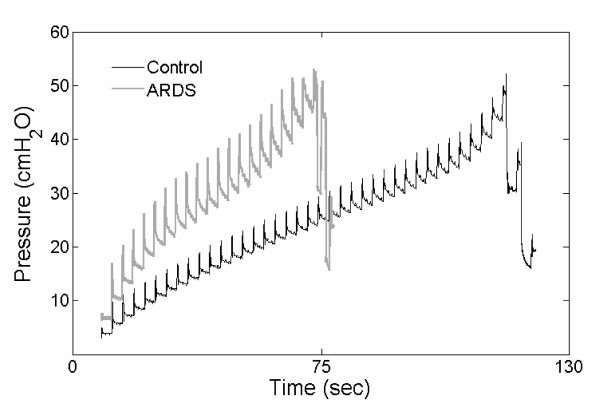
Super-syringe maneuver. Representative time-series for standardized super-syringe maneuvers obtained from one acute respiratory distress syndrome (ARDS) and one patient with healthy lungs (control). Volume steps of 100 mL were repetitively applied up to a maximum plateau pressure of 45 cmH_2_O. After each volume step, airflow was interrupted for three seconds.

Data were obtained from standardized super-syringe maneuvers [32] (Figure 1). Briefly, during the automatically operated maneuvers, the ventilator repetitively applied volume steps of 100 mL, with an inspiratory airflow rate of 558 ± 93 mL/sec for the ARDS group and 470 ± 95 mL/sec for the control group up to a maximum plateau pressure of 45 cmH_2_O. At the end of each volume application, airflow was interrupted for three seconds.

### Data analysis

All analyses and model simulations were carried out using the Matlab^® ^software package Version R2006b (The MathWorks^®^, Natick, MA, USA).

#### Model representation

**Figure 2:**
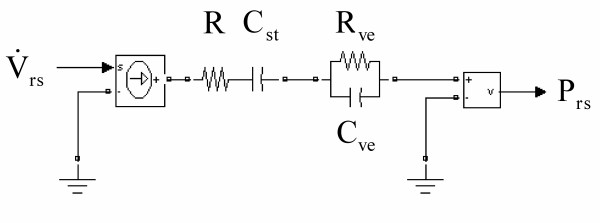
Lumped parameter model. Electrical circuit analog to the spring-and-dashpot model. R denotes the Newtonian airway resistance and C_st _the static compliance. R_ve _and C_ve _are the resistance and the compliance of the viscoelastic component, respectively. The respiratory airflow  represents the input and the respiratory pressure P_rs _the output of the model.

We used an electrical analog of a spring-and-dashpot model [19,21] (Figure 2) consisting of two components: (1) A Newtonian airway resistance (R) and a static compliance of the respiratory system (C_st_) and (2) the electrical analog of a resistive dashpot (R_ve_) and an elastic spring (C_ve_) as resistance and compliance of the component which is modeling viscoelastic behavior. The time constant of the viscoelastic component (τ_ve_) quantifies the stress relaxation dynamics of the system and is determined by the product of R_ve _and C_ve _[see Additional file 1].

#### Parameter estimation

**Figure 3:**
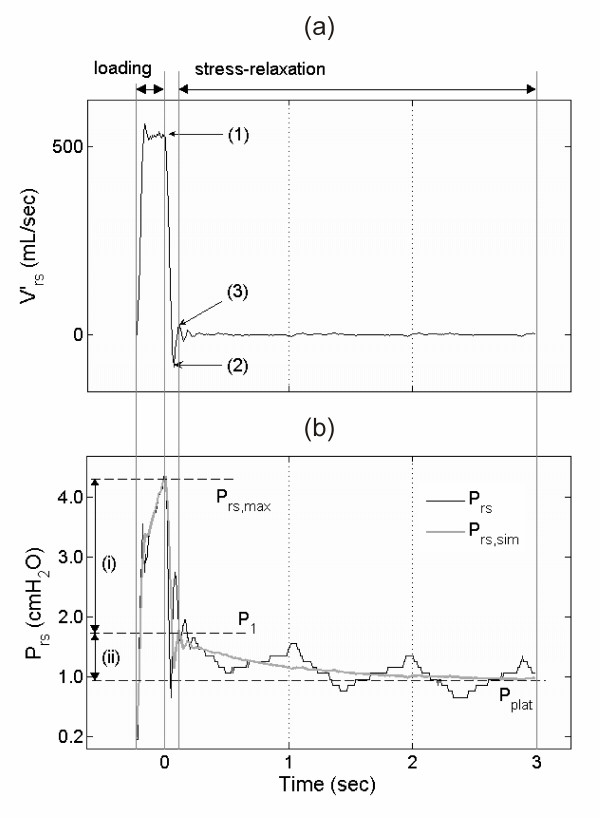
Flow interruption technique. **(a) **Respiratory flow  and **(b) **pressure P_rs _time-series of one 100 mL volume step including the phases of volume loading ( >0 mL/sec) and stress relaxation ( = 0 mL/sec during occlusion interval). (a) Labeled points indicate: (1) start of valve closure, (2) flow falling below zero due to valve characteristics, (3) estimated end of valve closure. The data between (1) and (3) were excluded from the fitting process [see Additional file 1]. (b) P_rs _with maximum pressure (P_rs, max_) and approximated plateau pressure (P_plat_). P_rs, sim _depicts the model-simulated respiratory pressure by use of the fitted parameter values. (i) denotes the initial resistive pressure drop (P_rs, max _down to P_1_), (ii) denotes the succeeding slow pressure change indicating stress relaxation between level P_1 _and P_plat_.

For each volume step i within each super-syringe maneuver, the parameters R^i^, C^i^_st_, R^i^_ve _and C^i^_ve_, were estimated by fitting the model via a multiple regression analysis to the time-series data (Figure 3) [see Additional file 1].

#### Impedance analysis

Impedance analysis was performed with respect to dependence on respiratory frequency for four categories: ARDS group at low (7.5 cmH_2_O) and high (42.5 cmH_2_O) plateau pressure, and control group at the same low and high plateau pressure. For each category, the parameters R, C_st_, R_ve _and C_ve _were determined and inserted into the model. For each parameterized model, a Bode magnitude plot was drawn.

#### Data presentation and statistical evaluation

For data presentation, the estimated values of the model parameters were linearly interpolated in steps of 2.5 cmH_2_O within a pressure range between 7.5 and 42.5 cmH_2_O. For each resulting pressure level, interpolated parameter values beyond the 1.5 fold of the interquartile range were eliminated as outliers. Normal distribution of the determined parameter values could not be proved. Therefore, statistical evaluation was based on the Wilcoxon rank-sum test. The significance level was set to *P *≤ 0.05. Data are presented as median (lower to upper quartile), unless otherwise indicated.

## Results

The super-syringe maneuvers consisted of 5 to 38 occlusions in the ARDS group, and 37 to 39 occlusions in the control group. The total inflated volumes were 1965 ± 929 mL for the ARDS group, and 4064 ± 67 mL for the control group.

**Figure 4:**
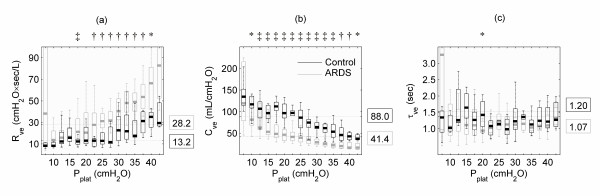
Results of parameter estimation. Estimated parameters of viscoelasticity for the acute respiratory distress syndrome (ARDS) group and the control group in terms of lower quartiles, medians and upper quartiles plotted against plateau pressure (P_plat_). Values on the right side of the diagrams indicate the overall medians. Statistically significant levels are indicated by * *P *≤ 0.05, † *P *≤ 0.01 and ‡ *P *≤ 0.001. **(a) **Resistance of viscoelastic model component (R_ve_) as well as **(b) **compliance of viscoelastic model component (C_ve_) differ significantly between both groups. For both parameters, a notably non-linear progression with increasing pressure was observed. **(c) **Time constant of viscoelastic model component (τ_ve_) does not differ between the two patient groups, and it does not depend on P_plat_.

Viscoelastic compliance, as well as viscoelastic resistance, depended on plateau pressure, and they differed between the control and ARDS groups. Viscoelastic resistance (Figure 4a) increased with pressure for both the control and the ARDS groups (control: 8.4 (7.4 to 11.9) up to 35.2 (25.6 to 39.5) cmH_2_O·sec/L; ARDS: 11.9 (9.2 to 22.1) up to 73.5 (56.8 to 98.7) cmH_2_O·sec/L). In contrast, viscoelastic compliance (Figure 4b) decreased with pressure for both groups (control: 130.1 (116.9 to 151.3) down to 37.4 (34.7 to 46.3) mL/cmH_2_O; ARDS: 125.8 (80.0 to 211.0) down to 17.1 (13.8 to 24.7) mL/cmH_2_O). Both interrelations presented a non-linear progression. At plateau pressures below 17.5 cmH_2_O, R_ve _remained almost constant with no significant differences between the control (10.1 (8.0 to 13.2) cmH_2_O·sec/L) and ARDS groups (12.8 (9.9 to 22.0) cmH_2_O·sec/L). At plateau pressures of 17.5 cmH_2_O and above, statistically significant differences were observed and increased with plateau pressure (control: 15.6 (10.7 to 26.6) cmH_2_O·sec/L; ARDS 34.7 (22.1 to 48.0) cmH_2_O·sec/L). In ARDS, the overall viscoelastic resistance was significantly larger (ARDS: 28.2 (15.4 to 42.9) cmH_2_O·sec/L; control: 13.2 (9.4 to 23.2) cmH_2_O·sec/L), and viscoelastic compliance was significantly smaller (ARDS: 41.4 (27.5 to 62.8) mL/cmH_2_O; control: 88.0 (64.0 to 111.8) mL/cmH_2_O) than control. In contrast, the viscoelastic time constant (Figure 4c) remained almost unchanged, and did not significantly differ between groups (ARDS: 1.07 (0.88 to 1.31) seconds, control group: 1.20 (0.92 to 1.58) seconds).

**Figure 5:**
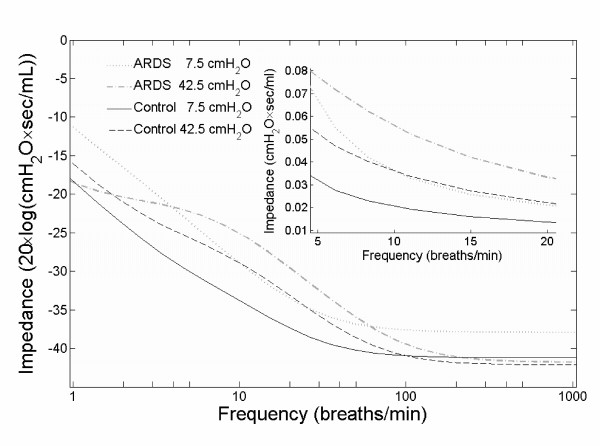
Frequency analysis. Frequency dependence of the respiratory systems mechanical impedance. The four curves were extracted from the magnitude diagram of a Bode plot, which was obtained from the Laplace transform representing the electrical circuit model. The curves represent the impedance of the model for plateau pressures of 7.5 cmH_2_O (low) and 42.5 cmH_2_O (high) for both patient groups. Note that the y-axis of the diagram is scaled by 20·log, i.e. dB, while the insert is linearly scaled.

With increasing respiratory frequency, the impedance of the respiratory system converged to a small value (Figure 5). At frequencies between 5 and 20 breaths/min, the respiratory system exhibited smaller impedances in control subjects compared with ARDS patients (Figure 5, insert). High plateau pressures induced higher impedance values than low pressures.

## Discussion

### Main findings

The main results of this study are: (i) The viscoelastic resistance R_ve _and compliance C_ve _depended non-linearly on increasing plateau pressure. In both groups, R_ve _increased and C_ve _decreased with increasing pressure, but these changes were different in ARDS and normal lungs. (ii) Stress relaxation dynamics represented by the time constant τ_ve _were independent of pressure and disease state (healthy vs. ARDS). (iii) The pulmonary mechanical impedance increased with plateau pressure and decreased with respiratory frequency.

### Mechanical properties

During each inflation step of a super-syringe maneuver, mechanical stress is applied to the lungs and part of the applied energy is loaded to the viscoelastic lung tissue components represented by the viscoelastic compliance, while part of this energy dissipates via the viscoelastic resistance. In the subsequent zero-flow phase the pulmonary tissue elements approximate a relaxation state at the new plateau pressure level. Therefore, each new inflation step starts from an increased baseline strain, which is quantified by the corresponding plateau pressure. Furthermore, each step starts from a particular relaxation state of the viscoelastic elements. Based on the fact that the pressure increase per 100 mL step of volume inflation was larger in ARDS, the super-syringe data revealed a discrepancy between groups concerning the number of volume steps. Despite these considerably different pressure volume relations, the time constant of viscoelasticity was independent of both, the pulmonary plateau pressure and also the disease state. Fung's [11] concept of quasi-linear viscoelasticity may provide a theoretical explanation: the experimental results showed that the quasi-static stress strain relation is non-linear [11,24]. On the other hand, stress relaxation dynamics are independent of strain. Implying quasi-linear viscoelasticity, Ingenito and colleagues [33] analyzed parenchymal tissue strips obtained from guinea pigs. They stated that in acute lung injury, changes in the elastic and dissipative properties of lung parenchyma can occur. Recently, Bates [24] transferred Fung's general mathematical concept to lung tissue mechanics by proposing a refined spring-and-dashpot model. This model is able to predict the stress relaxation power law in a strain-independent manner using a sequential recruitment of Maxwell bodies. The validation of the model was based on experiments with tissue strips taken from canine lung parenchyma [34]. The particular arrangement and interaction of the spring-and-dashpot elements of this model are well suited to describe viscoelastic tissue properties. Although using a basic lumped parameter model, our findings are consistent with Bates' observation [24] that his modeling approach exhibits quasi-linear viscoelastic behavior, in both qualitative and quantitative agreement with experimental data. Hence, the concept of quasi-linear viscoelasticity seems to apply to the human lung under mechanical ventilation: because the viscoelastic time constant τ_ve _was independent of the plateau pressure, stress relaxation was similar for all pressure levels. Referring to the non-linearity of the quasi-static stress strain relation, C_ve _and R_ve _showed distinct non-linear dependences on increasing plateau pressure (Figure 4). Compared with the normal lung, the increase in R_ve _in ARDS seemed to start at lower plateau pressures, and it had a steeper slope for pressures above 17.5 cmH_2_O. These findings are in accordance with previous studies [16,20] investigating the effect of PEEP on respiratory resistance. These studies found that the resistance is abnormally elevated in ARDS and that it increases with PEEP, particularly at 10 cmH_2_O and higher. This was assumed to be caused by stress adaptation phenomena and/or to be due to time constant inhomogeneities. Investigating data from patients with normal lungs, D'Angelo and colleagues [17] stated that the viscoelastic behavior of the lung is independent of volume and found no significant differences between ZEEP and PEEP (one level) for the viscoelastic parameters. Investigating data from ARDS patients, Eissa and colleagues [18] indeed found an increase of R_ve _and an increase of the viscoelastic elastance E_ve _(i.e. a decrease of C_ve _= 1/E_ve_) with an increasing PEEP level albeit a statistical significance could hardly be shown. The latter might stem from the rather small number of nine investigated subjects.

Concerning the pressure dependence of viscoelasticity, two situations can be distinguished: (i) in low pressure ranges, C_ve _is large and R_ve _is small; (ii) in high pressure ranges, the situation is reversed, with small C_ve _and large R_ve_. Therefore, in low pressure ranges, the viscoelastic compartment is characterized by a large loading capacity C_ve _for viscoelastic energy, which can easily dissipate via a small R_ve_. In contrast, at high pressure ranges, the situation is characterized by a small loading capacity C_ve_, and an impaired energy dissipation caused by a large R_ve_. During mechanical ventilation, low plateau pressure is therefore associated with small resistance imposed by the viscoelastic element, whereas at high plateau pressure, ventilation is impaired due to a large resistance generated by the viscoelasticity. Our results indicate that at low plateau pressures, that is below 20 cmH_2_O, viscoelastic resistance is not affected, whereas at high plateau pressures, it is. Therefore, within the context of the viscoelastic properties of lung tissue, ARDS patients might benefit from low alveolar pressure.

The difference between low plateau pressure (small R_ve_, large C_ve_) and high plateau pressure (large R_ve_, small C_ve_), as well as between ARDS and normal lungs, is responsible for the different sensitivity to respiratory frequency (Figure 5). The impedance values in the ARDS lungs at high plateau pressures exceeded impedance values in the normal lung at low plateau pressures by up to 270%. At higher frequencies, e.g. 60 to 900 breaths/min as used for high frequency oscillatory ventilation [35], the curves converged towards an identical small value. Hotchkiss and colleagues [6] observed in isolated rabbit lungs that ventilation at low respiratory frequencies caused less edema formation and histologic alterations than ventilation at high frequencies and identical tidal volume, airway plateau pressure, PEEP and peak pulmonary artery pressure. Interpreting these results from a mechanical-energetical point of view as underlying the present study, Hotchkiss and colleagues provided evidence that the amount of energy transfer indeed seems to be crucial for the induction of lung damage under mechanical ventilation: physically, the amount of mechanical energy is equivalent to the amount of mechanical work. This again is defined by the product of (volume-dependent) pressure and volume change. By keeping the applied pressure level and tidal volume constant, the transferred energy (energy per time) increases with increasing frequency, because over time the energy multiplies with the respiratory rate.

Thus, with respect to a clinical interpretation, from the mechano-energetical point of view our results might show evidence that ARDS patients would benefit from low alveolar pressures and high frequencies combined with reduced tidal volumes. If tidal volume is reduced under preservation of minute ventilation - in the context of protective ventilation - the same ventilatory effect can be achieved with a smaller energy transfer by a reduction of the frequency dependent impedance.

### Validity of method

For the present study, the data collection had to satisfy three main prerequisites: (1) to investigate stress relaxation dynamics, rapid flow interruptions were required; (2) the measured pressure-volume range had to be as wide as possible; and (3) a compromise between tolerable maneuver duration and desired high pressure/volume resolution had to be achieved. An appropriate measurement technique fulfilling these requirements was a standardized super-syringe maneuver [32]. Specifically, with respect to each of the prerequisites this method implies the application of flow interruptions, allowed for plateau pressures of up to 45 cmH_2_O, in our experimental conditions, and achieved a compromise by application of small 100 mL volume steps. In addition, due to the degree of automation and standardization of the technique, the super-syringe maneuvers were highly reproducible for all patients in both groups.

Pressure oscillations following the closure of the valve [36] are known to affect parameter estimation in the conventional two-point analysis [37-39]. Therefore, we excluded data corresponding to that time interval from the fitting and included, instead, the loading interval during volume inflation for the multiple regression analysis (Figure 3) [see Additional file 1]. This improved parameter estimation compared with the analysis exclusively based on the stress relaxation data. Specifically, the root mean squared error was reduced by 31% in the control group and by 55% in the ARDS group. A sensitivity analysis showed this approach to be very stable with respect to noise in the flow and pressure time series data.

In the literature [40,41], the side effects of prolonged closure of the occlusion valve on parameter estimation have been discussed. Corrections such as that of the maximal pressure at the end of the inspiratory flow phase have been suggested. However, these limitations do not apply to our experimental settings because the closure time of the ventilator valve we used was extremely short (1 ms, according to the manufacturer specifications).

Edibam and colleagues [42] found that during continuous tidal ventilation non-linear characteristics of the lung elastance depend on the flow pattern (pressure- vs. volume-control) and inspiratory to expiratory ratio. The differences in non-linear behavior were supposed to be most likely caused by the viscoelastic behavior of the respiratory system. Although the applied volume-dependent single-compartment model was well capable of describing the contribution of a non-linear elastance fraction to the total elastance of the respiratory system, it was not designed to quantify the potentially underlying viscoelastic effects. For this purpose a more expressive two-compartment model was applied in the present study. This model has been shown to adequately describe non-Newtonian behavior in normal lungs [19] and in ARDS at ZEEP [21]. In ARDS at PEEP, volume-dependent modeling of the viscoelastic compliance C_ve _improved the accuracy of the model[21]. Therefore, instead of direct non-linearity in the model itself non-linearity was approximated rather by a sequence of linear models parameterized on different pressure levels.

The question remains, if the data obtained with the 'static' super-syringe maneuver fits to respiratory mechanics during 'dynamic' continuous tidal ventilation. In contrast to classical analysis of static super-syringe maneuvers, we did not restrict our analysis to the equilibrated respiratory system (Figure 3b, P_plat_). Instead, we included the dynamic part during volume inflation to our model fit and focused on the dynamic stress relaxation response of the respiratory system during the zero-flow phase. Furthermore, with appropriate parameter settings the viscoelastic model has been shown to be independent of the flow pattern [19]. Taken together, we conclude that the estimated viscoelastic parameters are also valid during continuous tidal ventilation.

Gattinoni and Pesenti [2] showed that the ARDS lung is small rather than stiff and that at the same tidal volume, mechanical strain is larger in the small ARDS lung compared with the normal lung. Thus, given a small lung volume, even a low energy transfer may cause or aggravate ventilator-associated lung injury as the energy impacts on a smaller inner surface. Indeed, volumes applied within the super-syringe maneuvers were considerably smaller in ARDS (Figure 1). To prevent this bias the investigated mechanical parameters were interpreted in relation to respiratory pressure and not to lung volume.

### Limitations

Our study was designed retrospectively. The data have been previously published [25-27], yet the focus of the original reports was very different from that of the current study. As one of the maneuvers in the collection of data was a standardized super-syringe maneuver, which was our method of choice, and the data had been measured independently as part of two different studies, the available data was ideal for our experiments. Furthermore, conducting additional patient measurements when such a pool of data was available would not have been reasonable, particularly as the super-syringe data had not been previously examined in the context of the problem discussed here [25].

The effects of viscoelasticity and pendelluft (volume equilibration between compartments of the inhomogeneous lung) are hard to distinguish. Therefore, although we used a model that has been proven to be appropriate to study viscoelastic behavior in the homogeneous, healthy lung [43], we are being very careful in our interpretations, following the example of preceding studies in this field.

Due to the mechanical inhomogeneity of the ARDS lung there is a particular dilemma when healthy lungs are compared with ARDS lungs. Because of alveolar consolidation and atelectasis, inflation with constant volume steps of 100 mL is in all likelihood delivered to a smaller amount of lung tissue in ARDS than in normal controls. This aggravates the interpretation of our findings from a physiological point of view. However, this does not impact the clinical implications of limiting the energy transfer to the lung.

Low plateau pressure values were hard to observe in ARDS. This is likely to be due to the high opening pressures of the ARDS lung. In addition, in the control group, high pressure ranges were not frequently measured because this was prevented by a built-in safety feature in the device. Therefore, parameters could rarely be estimated for low (ARDS) and high (control) pressures, which resulted in high variances for the parameters estimated within these ranges.

## Conclusions

To the best of our knowledge this is the first study investigating the viscoelastic resistance, compliance and time constant on data covering the whole range of inspiratory capacity. Non-linear pressure-dependencies of the lung viscoelasticity differ between patients with healthy and ARDS lungs. In contrast, the time constants of stress relaxation processes are independent of pressure and respiratory disease. These findings confirm Fung's concept of quasi-linear viscoelasticity. Finally, the impedance of the respiratory system interacts with its viscoelastic properties. With regard to clinical evidence, we cautiously conclude that by application of low inspiratory pressures and high respiratory frequencies combined with low tidal volumes, the energy transfer from the respirator to the lung can be reduced. This in turn is potentially lung-protective.

## Key messages

• Resistive and elastic components of pulmonary viscoelasticity analyzed in stress relaxation processes in the mechanically ventilated human lung are highly non-linear and depend on pressure.

• Resistive and elastic components of pulmonary viscoelasticity differ between normal and ARDS lungs whereas the viscoelastic time-constant does not.

• From the aspect of a reduced energy transfer from the respirator to the patient's lung, the analysis of pulmonary viscoelasticity seems to affirm the mechanical ventilation approach with low inspiratory pressure, high respiratory frequency and low tidal volume as lung protective.

## Abbreviations

ARDS: acute respiratory distress syndrome; ASA: American Society of Anesthesiologists' physical status; C_st_: static compliance; C_ve_: compliance of viscoelastic model component; FiO_2_: fraction of inspired oxygen; PEEP: positive end-expiratory pressure; R: Newtonian airway resistance; R_ve_: resistance of viscoelastic model component; τ_ve_: time constant of viscoelastic model component; ZEEP: zero end-expiratory pressure.

## Competing interests

The authors declare that they have no competing interests.

## Authors' contributions

SG designed the study, preprocessed and analyzed the data and wrote the manuscript. JG and KM assisted study design, data analysis and writing. DS participated in data measurement and assisted writing. SS assisted data analysis and writing.

## Supplementary Material

Additional file 1 PDF file that includes details of the data preprocessing and the multi-regression analysis. Click here for file
