# Supplementary material for: Pressure-dependent stress relaxation in acute respiratory distress syndrome and healthy lungs: an investigation based on a viscoelastic model
Source: Crit Care. 2009 Dec 9;13(6):R199. doi: 10.1186/cc8203 (PMC2811954; doi:10.1186/cc8203)
Supplement: Additional file 1 — PDF file that includes details of the data preprocessing and the multi-regression analysis. [file cc8203-S1.PDF]

Data preprocessing

To prevent data analysis from being affected by the pressure oscillations caused by the closure of the interruption valve, the referring data interval was excluded from the data fitting. The time period corresponding to the valve-induced pressure oscillations was defined as the interval between the onset of flow interruption and the end of the first positive slope of the $\dot{V}_{rs}$ curve (Fig. 3).

It was assumed that the stress-relaxation was completed after a 3 sec occlusion interval, and that by then, pressure had reached P_{plat}. Thus, the respiratory pressure of the i-th volume step (P^{i}_{rs}) was adjusted with an offset determined by the plateau pressure P^{i-1}_{plat} of the preceding maneuver volume step i-1 (Equation 1, Fig. 3).

$$P_{rs}^{i}(t) = P_{rs} - P_{plat}^{i-1} \tag{1}$$

P_{plat} was calculated as the mean of the pressure values obtained from a 0.5 seconds interval at the end of occlusion. For step i = 1, P^{0}_{plat} was set to the mean of the last 0.5 seconds of the preceding expiration at ZEEP.

Multi-regression analysis

Representation of the electrical circuit (Fig. 2) in terms of a Laplace transform leads to Equation 2.

$$\frac{P_{rs}(s)}{\dot{V}_{rs}(s)} = \frac{RC_{st}R_{ve}C_{ve}s^{2} + (RC_{st} + C_{st}R_{ve} + R_{ve}C_{ve})s + 1}{C_{st}R_{ve}C_{ve}s^{2} + C_{st}s} \tag{2}$$

where the pressure of the respiratory system (P_{rs}) is represented by the numerator, and the respiratory flow ($\dot{V}_{rs}$) by the denominator. The sum of squared errors was used as the scalar function of R, C_{st}, R_{ve} and C_{ve} to be optimized. For optimization the simplex search method of Lagarias et al. [44] was used.
